# Stage-specific changes in schematic facial emotion recognition following childhood trauma: an ERP study

**DOI:** 10.1186/s40359-026-05135-8

**Published:** 2026-07-21

**Authors:** Siyu Chen, Danmin Miao

**Affiliations:** Department of Military Medical Psychology, Air Force Medical University, Xi’an, 710032 China

**Keywords:** Childhood trauma, Schematic faces, Emotional face processing, ERP

## Abstract

**Background:**

Childhood trauma has been linked to emotional face processing, but its association with stages of emotional face processing remains unclear. The present study used schematic emotional facial cues as simplified stimuli and event-related potentials to examine early face sensitive activity and later evaluative processing within this stimulus context.

**Methods:**

Adults with and without childhood trauma completed an emotion classification task with happy, sad, and neutral schematic faces during EEG recording. Reaction times and accuracy were analyzed as behavioral measures. ERP analyses focused on the N170 and VPP in the early window and the P300/LPP complex in the later window.

**Results:**

The childhood trauma group showed longer reaction times than the control group, with no significant difference in accuracy. In the early window, the childhood trauma group showed earlier N170 but later VPP peak timing. Early amplitude patterns also differed, with posterior N170 sad–happy differentiation in controls and happy-dominant VPP modulation in the childhood trauma group. In the later window, the childhood trauma group showed overall larger overall P300/LPP amplitudes and longer latencies than the control group. P300/LPP amplitude also varied by group and emotion, with a sad-over-happy pattern in the childhood trauma group and a happy-over-sad pattern in controls.

**Conclusions:**

Childhood trauma was associated with slower responding and stage-specific ERP differences during schematic emotional face processing. These differences appeared across both the early N170, VPP and later P300/LPP windows, rather than as a uniform change in responsiveness. The findings suggest altered timing and emotion-specific weighting across ERP windows, including enhanced late positive responses to sad relative to happy schematic faces in the childhood trauma group.

## Introduction

Facial emotion recognition is a core component of social functioning. By identifying emotional signals conveyed by facial cues, individuals can infer others’ emotional states and adjust their responses during social interaction. Childhood maltreatment has been associated with later difficulties in emotion understanding, empathy, and social functioning [1], and altered facial emotion recognition performance is commonly reported in individuals with histories of early adversity [2]. Childhood trauma commonly refers to highly distressing developmental experiences that overwhelm an individual’s coping capacities, including sexual abuse, physical abuse, emotional abuse, and neglect [3]. Behavioral findings suggest that trauma-related differences in facial emotion recognition are mixed rather than uniform. Some studies point to heightened sensitivity or preferential responses to negative or threat-related facial cues, particularly anger and fear [4–6]. Other findings indicate that these differences are not restricted to negative expressions and may also involve altered processing of happy or emotionally salient faces [7]. These mixed behavioral patterns may reflect variation across emotional expression, response index, task context, and sample characteristics. Behavioral studies typically rely on accuracy and reaction time to characterize recognition performance. These measures summarize the final behavioral response and cannot determine whether trauma-related differences emerge during early face-sensitive processing, later evaluative processing, or across these processing periods. A temporally sensitive neural measure is therefore needed to clarify how childhood trauma is associated with the unfolding of facial emotion processing before the final behavioral response.

Event-related potentials provide a suitable method for addressing this question because they can track neural responses with millisecond-level temporal resolution [8]. In emotional face processing, ERP studies commonly distinguish early face-sensitive responses from later evaluative responses to emotional significance [9–11]. This temporal distinction is relevant to childhood trauma because adverse caregiving environments have been proposed to recalibrate sensitivity to socially salient facial cues, especially cues associated with potential threat or rejection [12–14]. Such trauma-related differences may be expressed in early sensitivity to emotional facial cues, in later evaluation of their emotional significance, or across these selected processing periods. The present study used this framework to examine early face-sensitive processing and later evaluative processing during schematic facial emotion recognition. Early face-sensitive ERP evidence provides a more specific basis for examining the N170 response in the present study. In childhood maltreatment and interpersonal-trauma samples, N170 findings have involved both enhanced amplitudes to emotional faces and altered differentiation between threat-related and positive facial cues [15, 16]. This evidence links N170 modulation to the question of whether childhood trauma is associated with altered early emotion-sensitive differentiation during facial emotion processing. The VPP was examined as a distinct fronto-central component in the same early processing period because this positive response is closely related to face-sensitive neural activity and may provide complementary information about early responses to schematic emotional face [17]. The later processing period is relevant because maltreatment-related ERP studies have also reported altered late positive responses to emotional facial cues. Such effects have been observed in maltreated children and in adults with childhood emotional abuse histories [18, 19]. These findings suggest that childhood trauma may also be associated with later evaluative responses to emotional facial information. Existing ERP findings remain difficult to integrate because studies differ in stimulus format, task demands, trauma or adversity type, participant age, and the ERP components used to characterize processing. It therefore remains unclear whether trauma-related differences in early face-sensitive responses are accompanied by differences in later evaluative processing within the same facial emotion recognition task.

Stimulus format may influence the interpretation of trauma-related differences in facial emotion processing. Photographic facial stimuli preserve richer social and perceptual information than schematic faces, but they also retain identity-related features that are not central to the present research question. Even when photographic face sets are standardized, they contain information about identity, age, gender, ethnicity, skin texture, and other physical facial characteristics. Such physical variability can influence early face-sensitive neural responses and may complicate the interpretation of trauma-related effects [20]. Social perception can be shaped by identity-related and contextual cues, including dress and other appearance-based information [21]. Trauma-learning models further suggest that otherwise neutral cues can acquire emotional or threat-related significance after aversive experience [22]. In studies of childhood trauma, such information may add sources of variation that are difficult to separate from responses to emotional facial configurations. The present study used schematic emotional faces to reduce identity-related and social-contextual variability and to narrow the analysis to simplified emotional facial configurations. Prior ERP evidence indicates that schematic faces can elicit early face-sensitive responses, including N170 activity [23]. This choice reduces variability arising from identity-related and contextual cues, but it also limits ecological validity and narrows the scope of interpretation. Findings based on schematic emotional faces should be generalized cautiously to facial emotion recognition involving photographic or real-world facial expressions. The present study followed an ERP framework that differentiates early face-sensitive perceptual encoding from later evaluative processing. Accordingly, we examined the N170, VPP window and the P300/LPP complex to characterize whether trauma-related neural differences were concentrated in early face-sensitive processing, extended to later evaluative processing, or involved coordinated changes across these processing periods. Specifically, the N170, a negative-going component observed over occipito-temporal sites approximately 140–200 ms after stimulus onset, is commonly associated with early perceptual and face-structural encoding [9]. The VPP is a positive going fronto-central component occurring within a similar early latency range and is closely linked to the N170 as another expression of early face-related neural activity [17]. The P300/LPP complex is typically interpreted as reflecting later evaluative processing, attentional resource allocation, and context updating [10, 11]. Although P300 and LPP are sometimes treated as distinct components, they are considered here as a unified index of later evaluative processing given their temporal overlap and shared sensitivity to emotional significance.

Based on the rationale outlined above, hypotheses were formulated for behavioral performance, N170 responses, VPP responses, and the later P300/LPP complex. At the behavioral level, prior findings have linked childhood trauma and adversity to altered facial emotion recognition performance, including prolonged reaction times in young adults with childhood adversity [24]. Participants with childhood trauma were therefore expected to show reduced efficiency in emotional face recognition, reflected primarily in longer reaction times relative to controls. Prior work also suggests that childhood trauma and maltreatment may alter sensitivity to negative social-emotional cues [12, 13]. The trauma group was expected to show altered emotional modulation of reaction time, with stronger slowing for negative emotional faces than for happy or neutral faces. Because prior behavioral findings have varied across emotion categories, task demands, and sample characteristics, no strong directional prediction was formulated for accuracy. For the early N170 response, prior ERP studies suggest that childhood trauma and maltreatment are related to altered early face-sensitive responses during emotional face processing [15, 16]. The threat vigilance account further suggests that adverse caregiving environments may recalibrate early sensitivity to socially relevant emotional cues, especially cues associated with potential threat or rejection. Based on this evidence, the childhood trauma group was expected to show stronger N170 modulation for negative emotional configurations than for happy or neutral configurations. N170 latency was also examined as a complementary index of the timing of early face-sensitive processing. Because prior findings provide less consistent evidence for the direction of trauma-related latency effects, no strong directional prediction was made for N170 latency. The VPP was examined as a distinct fronto-central index of early face-related neural activity. Because direct evidence linking childhood trauma to VPP responses during facial emotion recognition remains limited, the VPP hypothesis was formulated more cautiously than the N170 hypothesis. Group differences in VPP emotional modulation were examined without specifying a strong directional prediction. For the later P300/LPP complex, the prediction focused on the amplitude of later evaluative responses to emotional faces. Maltreatment-related ERP evidence indicates that late positive activity can be altered during emotional face processing, with effects reported in both maltreated children and adults with childhood emotional abuse histories [18, 19]. Because these findings do not establish a single direction of late positive activity across samples and stimulus contexts, the P300/LPP amplitude prediction was formulated cautiously. The childhood trauma group was tentatively expected to show reduced P300/LPP amplitudes to negative emotional faces. Across ERP measures, childhood trauma was expected to be associated with component- and window-dependent emotional modulation across N170, VPP, and P300/LPP responses.

## Method

### Participants

The Childhood Trauma Questionnaire–Short Form [25] was administered to screen 500 undergraduates at Shaanxi Normal University. Data were collected using an online survey. The survey was distributed on the Questionnaire Star platform (https://www.wjx.cn/). Forty-four participants were recruited, being divided into two groups: a childhood trauma group (*n* = 22; 10 male, 12 female; M age = 19.16, SD = 0.76) and a matched control group (*n* = 22; 10 male, 12 female; M age = 19.05, SD = 0.40). The total sample included 20 males and 24 females. All participants were 18 to 20 years old. Exclusion criteria: left-handedness; history of brain injury; diagnosis of neurological or psychiatric disorders; or uncorrected visual impairments. Both groups were matched on age and sex. The study was approved by the Ethics Review Committee of City University of Macau. All participants provided written informed consent and were informed that they could withdraw at any time without penalty. Further details can be found in Table 1.

**Table 1:** Age and CTQ-SF scores in the trauma and control groups

Group	Age (years)						CTQ-SF score				
	Min	Max	Mean ± SD	t	*p*		Min	Max	Mean ± SD	t	*p*
Trauma	18	20	19.16 ± 0.76	0.53	0.600		40	60	54.68 ± 5.67	8.27	< 0.001
Control	18	20	19.05 ± 0.40				25	30	26.74 ± 1.82		

The statistical results (p-values and t-values) were obtained using independent samples t-tests between Trauma and Control group

*CTQ-SF* Childhood Trauma Questionnaire–Short Form

### Measures

#### Childhood trauma questionnaire -short form

Childhood trauma was assessed with the Chinese version of the Childhood Trauma Questionnaire–Short Form [25]. The CTQ-SF contains 28 items rated on a 5-point Likert scale (1 = never true to 5 = very often true) and yields five 5-item subscales—Emotional Abuse, Physical Abuse, Sexual Abuse, Emotional Neglect, and Physical Neglect. The CTQ-SF also included three denial items. Subscale scores are summed (range 5–25), with higher scores indicating more severe maltreatment. The study used severity cutoffs [3] for moderate to severe levels. The cutoffs were as follows: Emotional Abuse ≥ 9, Physical Abuse ≥ 10, Sexual Abuse ≥ 6, Emotional Neglect ≥ 15, Physical Neglect ≥ 10. In this study, the overall Cronbach’s alpha coefficient was 0.85.

Participants were placed in the childhood trauma group if at least one CTQ-SF subscale score met the moderate to severe cutoff. The researcher selected the control group from participants with low CTQ-SF scores. The control group was matched to the childhood trauma group on age and sex. Researchers also checked the denial scale to reduce the risk of inaccurate reporting on the CTQ-SF. The research team then conducted semi-structured interviews with each participant. Semi-structured interviews were subsequently conducted with each participant to confirm group eligibility and to verify the nature and context of reported traumatic experiences.

To mitigate sample heterogeneity, a multi-stage screening procedure was implemented. Childhood trauma group membership required meeting moderate-to-severe cutoffs on at least one CTQ-SF subscale. The denial scale was examined and semi-structured interviews were conducted with each participant to confirm eligibility and reduce misclassification. Both groups were matched on age and sex.

#### Schematic faces

**Fig. 1:**
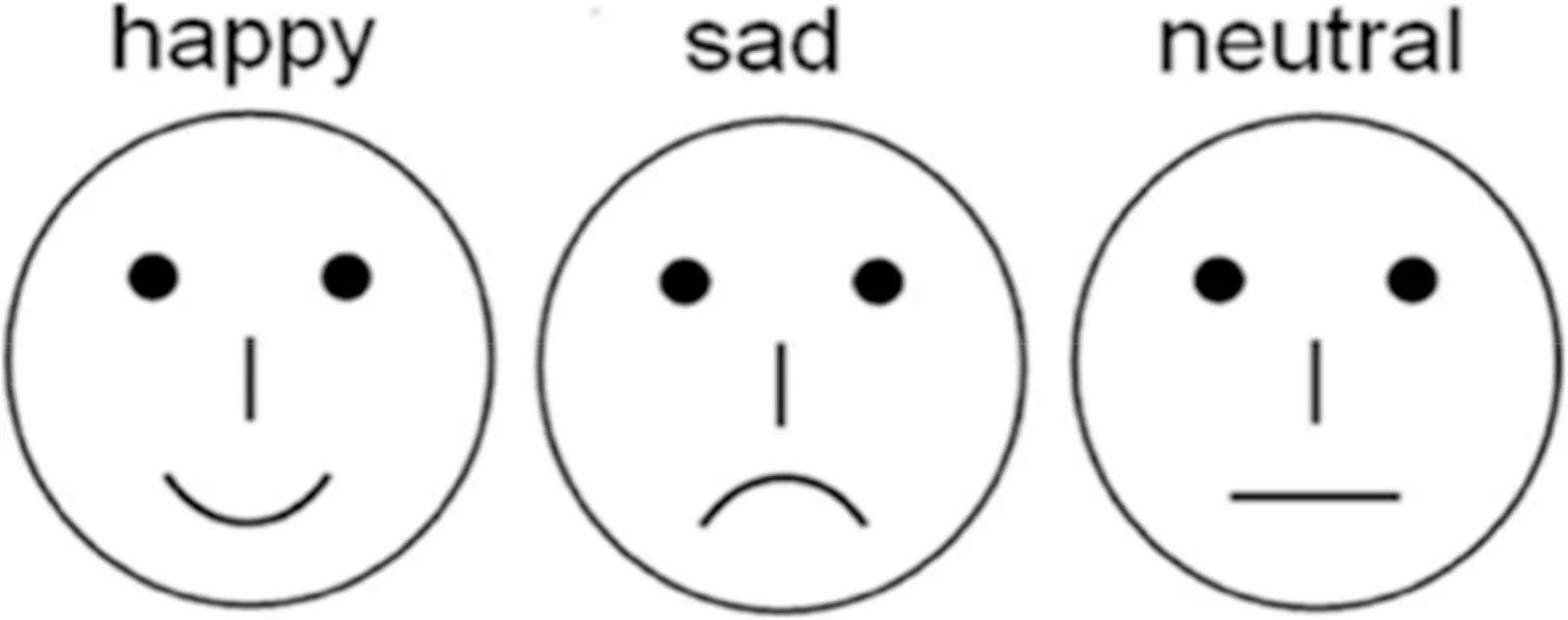
Schematic face stimuli used in the experiment

We examined how childhood trauma affects emotional information processing. Photo-based face stimuli can vary in ethnicity, gender, age, facial identity, and emotional intensity, which may introduce uncontrolled perceptual and identity-related variability. For this reason, we used standardized schematic facial expressions as the experimental stimuli. Prior neuroimaging research indicates that schematic faces convey core affective information while reducing interference from complex physical features [26], and they have been used to probe emotion-related neural mechanisms; studies report engagement of the amygdala and prefrontal cortex [27]. The present stimuli, illustrated in Fig. 1, consisted of three schematic emotional expressions: happy, sad, and neutral. These categories were selected because they have been used in previous ERP research on schematic emotional face processing [28]. Happy and sad faces were included to provide simplified positive and negative emotional cues, respectively, whereas neutral faces served as a non-emotional comparison condition. Third, limiting the paradigm to three conditions reduced task burden and helped maintain a sufficient number of trials per condition for reliable ERP averaging. All images were processed to maintain a constant size and were presented using E-Prime 3.0 on an LCD monitor with a resolution of 1024 × 768 pixels and a refresh rate of 60 Hz. Stimuli were displayed centrally on a mid-gray background, and participants viewed them from a distance of 60 cm.

### Experimental procedure

**Fig. 2:**
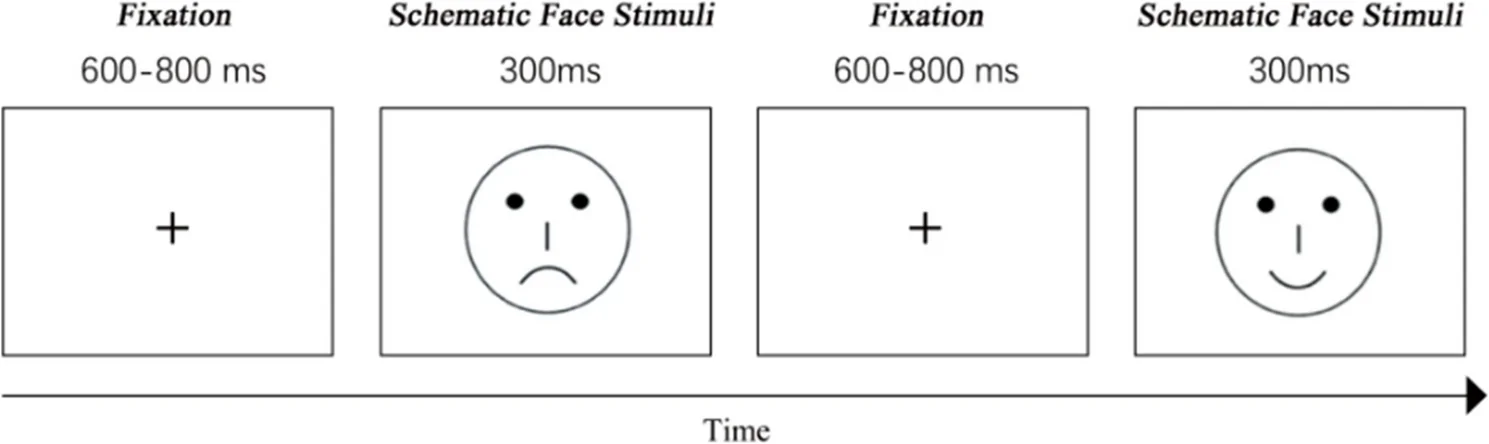
Experimental flowchart of the trial procedure. Each trial began with a fixation cross presented for 600–800 ms, followed by a schematic face stimulus displayed for 300 ms. A total of 300 trials were administered, comprising 100 trials for each facial expression condition. Keypress assignments were counterbalanced across participants

The experiment took place in a quiet, soundproof room with a comfortable ambient temperature. Stimuli were presented in a pseudo-random order using the E-Prime 3.0 program. After the informed consent, they performed a facial-emotion categorization task with a keypress. Before the experiment, each participant underwent a training session to familiarize themselves with the procedures. Participants were instructed to judge as accurately and quickly as possible whether the face was happy, sad, or neutral by pressing a key (“z”, “x”, or “c”). At the beginning of each trial, a fixation cross appeared on the screen for a duration ranging from 600 ms to 800 ms. Subsequently, a face was presented for 300 ms. When participants failed to respond, their responses were counted as errors in the accuracy metric and were excluded from reaction-time analyses. Each subject will be randomly presented with 300 trials, comprising 100 trials for each of the three facial expressions. Keypressing was counterbalanced across participants to control for potential order effects. The experimental procedure is summarized in Fig. 2.

Each trial began with a fixation cross presented for 600–800 ms, followed by a schematic face stimulus displayed for 300 ms. A total of 300 trials were administered, comprising 100 trials for each facial expression condition. Keypress assignments were counterbalanced across participants.

### EEG recording and processing

EEG was recorded with a wireless 32-channel Quick-30 headset (CGX/Cognionics, San Diego, CA, USA) sampled at 500 Hz. Electrodes were referenced to the right mastoid (A2) during recording. Non-EEG auxiliary channels (ExG1, ExG2) and accelerometer channels (ACC32, ACC33, ACC34) were removed prior to analysis. Electrode impedances were maintained below 5 kΩ for all sensors. Preprocessing was performed in EEGLAB (version 2021.0) running on MATLAB R2022b. Noisy EEG channels were identified using automated channel statistics implemented in EEGLAB and visual inspection, then reconstructed with spherical-spline interpolation in EEGLAB. On average, 0.8 ± 0.8 channels were interpolated per participant in the childhood trauma group, and 0.7 ± 0.7 channels were interpolated per participant in the control group. No participant had more than 2 interpolated channels. Interpolation was limited to a small number of isolated sensors and did not affect regions of interest. After channel interpolation, the continuous EEG data were re-referenced offline to the average of all retained scalp electrodes; A2 thus served solely as the online recording reference, and all subsequent ERP analyses were conducted on average-referenced data. Data were decomposed using Independent Component Analysis, and components reflecting blinks, saccades, and cardiac activity were removed. Data were segmented into epochs from − 200 to 800 ms time-locked to the onset of facial expressions. Baseline correction used the − 200 to 0 ms interval. For ERP averaging, trials with missing behavioral responses were excluded automatically because they did not contain valid response codes. Given the extremely small proportion of such trials, their exclusion had a negligible impact on the number of artifact-free epochs retained per condition. Artifact-free epochs were averaged separately for happy, neutral, and sad conditions. Finally, extracted component-level mean-amplitude and peak-latency values were inspected for extreme observations, and no values requiring exclusion were identified.

Following conversion to the average reference, ERP components were re-examined using grand-averaged waveforms collapsed across groups and conditions, together with their topographic distributions. In the present study, three ERP components were analyzed for each participant: N170, VPP, and P300/LPP complex. To increase the signal-to-noise ratio and focus on theoretically relevant regions of interest, ERP measures were quantified using predefined, literature-informed electrode clusters [8]. For the VPP, measures were extracted from a fronto-central cluster comprising Fz and Cz, consistent with prior work describing the VPP as a vertex/fronto-central positivity that accompanies the N170, occurs within a similar latency range, and is closely linked to early face-related processing [17, 29]. For P300/LPP complex, measures were extracted from a centro-parietal cluster comprising Cz and Pz [10, 11]. The predefined time windows were 150–200 ms for the N170, 150–250 ms for the VPP, and 320–600 ms for the P300/LPP complex. The predefined time windows were 150–200 ms for the N170, 150–250 ms for the VPP, and 320–600 ms for the P300/LPP complex. Although the N170 and VPP occurred in a partially overlapping early latency range, they were quantified separately because they were maximal over distinct posterior and fronto-central ROIs and showed separable scalp distributions. ERP measurement windows and ROIs were selected based on prior ERP literature and verified using the average-referenced collapsed grand-average waveforms across groups and emotional conditions. The collapsed grand-average waveforms showed that the N170 peaked at approximately 174 ms over both left and right posterior ROIs, within the 150–200 ms analysis window. The VPP peaked at approximately 184 ms over the Fz/Cz ROI, within the 150–250 ms window. The P300/LPP complex, quantified over the Cz/Pz ROI, peaked at approximately 388 ms, within the 320–600 ms window. These grand-average peak latencies were used to verify the suitability of the measurement windows; statistical analyses were conducted on participant-level mean-amplitude and peak-latency values extracted automatically using MATLAB. For each participant and condition, mean amplitude was calculated by averaging voltage values across the corresponding electrode cluster and time window. Peak latency was defined as the time point of the maximum negative peak for the N170 and the maximum positive peak for the VPP and P300/LPP complex within the corresponding time window. The ERP figures were redrawn using the average-referenced data, and the shaded time windows correspond to the final component quantification windows.

### Data analysis

Reaction times and accuracy were analyzed using mixed repeated-measures ANOVAs, with emotion (happy, sad, neutral) as the within-subject factor and group (control, childhood trauma) as the between-subjects factor. For behavioral analyses, non-response trials were defined as trials without a valid button response within the response window. These trials were excluded from reaction-time analyses and were counted as incorrect responses when calculating accuracy. Non-response rates were calculated separately for each participant and condition and were analyzed using the same mixed repeated-measures ANOVA structure to determine whether missed responses differed between groups or emotional conditions. For the ERP measures, mean amplitudes and peak latencies of the N170, VPP, and P300/LPP complex were entered into mixed repeated-measures ANOVAs. For the N170, emotion (happy, sad, neutral) and hemisphere (left, right) were included as within-subject factors, and group (control, childhood trauma) was included as the between-subjects factor. For the VPP and P300/LPP complex, mean amplitude and peak latency values were extracted from waveforms averaged across the predefined electrode clusters before statistical analysis. Separate mixed-design ANOVAs were then conducted with emotion (happy, sad, neutral) as the within-subject factor and group (control, childhood trauma) as the between-subjects factor. As part of the final data verification procedure, participant-level N170 mean amplitude values were screened for extreme values within each group-by-emotion-by-hemisphere cell. Values exceeding ± 3 standard deviations from the corresponding cell mean were defined as extreme values and were checked against the original ERP waveforms and preprocessing records before exclusion. Degrees of freedom for the F tests were adjusted using the Greenhouse–Geisser correction when appropriate. Statistical significance was set at *p* < .05, and post hoc p values were Bonferroni-corrected for multiple comparisons. Statistical analysis was performed using SPSS 29 (IBM Corp., Armonk, NY, USA).

## Results

### Behavioral results

Behavioral measures across the three emotion conditions (happy, neutral, and sad) in the two groups are presented in Table 2.

**Table 2:** Summary of statistical results for behavioral data

Group	Reaction time (ms)			Accuracy(%)		
	happy	neutral	sad	happy	neutral	sad
Childhood trauma	589.52 ± 61.65	525.11 ± 60.35	560.05 ± 46.49	96.96 ± 1.46	96.75 ± 1.43	97.27 ± 1.18
Control	517.31 ± 55.48	493.97 ± 60.47	506.99 ± 57.20	97.54 ± 1.99	97.68 ± 1.75	97.31 ± 1.20

Descriptive statistics (mean ± SD) for reaction time, accuracy in each condition are presented in Table 2

A significant between-group difference in reaction times was observed, *F*(1, 42) = 12.53, *p* = .001, ηp² = 0.230, with the childhood trauma group (*M* = 558.22 ms, *SD* = 45.99 ms) responding more slowly than the control group (*M* = 506.09 ms, *SD* = 51.55 ms). The main effect of emotion was also significant, *F*(2, 84) = 16.05, *p* < .001, ηp² = 0.276. This effect was qualified by a significant emotion-by-group interaction, *F*(2, 84) = 3.51, *p* = .034, ηp² = 0.077. Bonferroni-adjusted p values are reported for all follow-up comparisons. Group comparisons within each emotion showed that the childhood trauma group responded more slowly than the control group for happy faces (childhood trauma group: *M* = 589.52 ms, *SD* = 61.65 ms; control group: *M* = 517.31 ms, *SD* = 55.48 ms; Δ = 72.21 ms, adjusted *p* = .001) and sad faces (childhood trauma group: *M* = 560.05 ms, *SD* = 46.49 ms; control group: *M* = 506.99 ms, *SD* = 57.20 ms; Δ = 53.06 ms, adjusted *p* = .005). The group difference for neutral faces was not significant (childhood trauma group: *M* = 525.11 ms, *SD* = 60.35 ms; control group: *M* = 493.97 ms, *SD* = 60.47 ms; Δ = 31.14 ms, adjusted *p* = .284). Analyzed separately by group, the emotion effect was not significant in the control group, *F*(2, 42) = 2.96, *p* = .063, ηp² = 0.124. In contrast, the childhood trauma group showed a significant effect of emotion, *F*(2, 42) = 14.04, *p* < .001, ηp² = 0.401. Bonferroni-adjusted comparisons showed that the childhood trauma group responded more slowly to happy faces than neutral faces (Δ = 64.41 ms, adjusted *p* < .001) and more slowly to sad faces than neutral faces (Δ = 34.94 ms, adjusted *p* = .010). Happy and sad faces did not differ significantly in the childhood trauma group (Δ = 29.47 ms, adjusted *p* = .073). Accuracy was analyzed using the same 2 × 3 mixed repeated-measures ANOVA. The main effect of group was not significant, *F*(1, 42) = 2.92, *p* = .095, ηp² = 0.065, indicating that accuracy did not differ reliably between the childhood trauma group (*M* = 96.99%, *SD* = 0.84%) and the control group (*M* = 97.51%, *SD* = 1.14%). The main effect of emotion was also not significant, *F*(2, 84) = 0.03, *p* = .970, ηp² = 0.001. Accuracy was comparable across happy faces (*M* = 97.25%, *SD* = 1.75%), neutral faces (*M* = 97.22%, *SD* = 1.65%), and sad faces (*M* = 97.29%, *SD* = 1.18%). The emotion-by-group interaction was not significant, *F*(2, 84) = 1.11, *p* = .336, ηp² = 0.026. Thus, the behavioral group differences observed in reaction times were not accompanied by corresponding accuracy differences. Non-response trials were extremely rare, accounting for only 0.17% of all behavioral trials. Only 23 non-response trials were observed across the entire dataset, involving three participants: two in the control group and one in the trauma group. These missed responses were distributed across happy, sad, and neutral trials as 10, 9, and 4 trials, respectively. Given the floor-level frequency of non-responses, no separate inferential analysis was conducted.

### Electrophysiological results

**Fig. 3:**
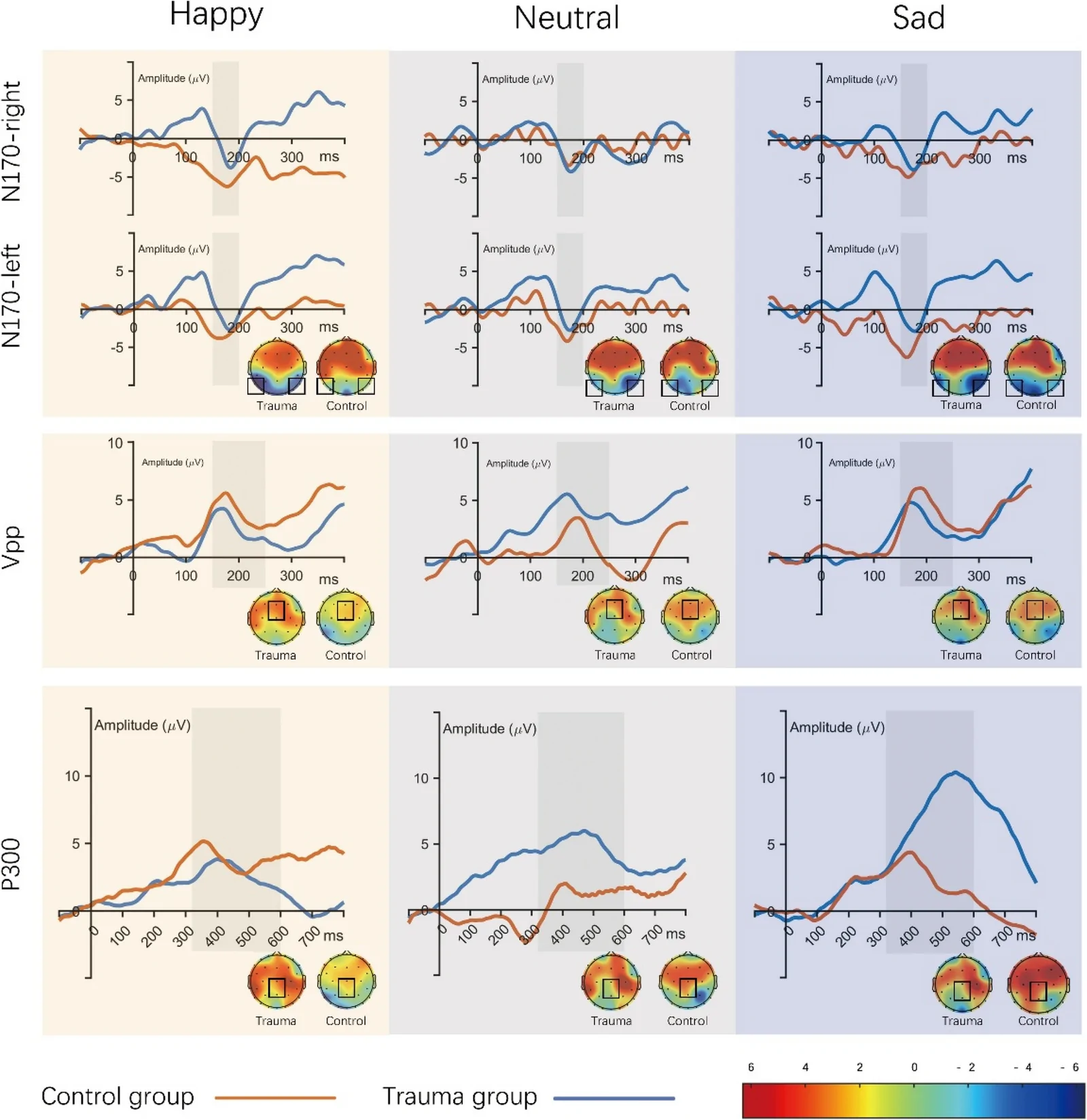
ERP responses in the experiment. Grand-average ERP waveforms for the control group (red) and childhood trauma group (blue) across the selected ROI shown for schematic emotional faces in the happy (yellow background), neutral (gray background), and sad (blue background) conditions. Scalp topographies of the primary ERP components (N170,VPP, and P300/LPP complex) are shown at the component-specific peak latencies identified within the predefined time windows. Electrode sites used to quantify component amplitudes are indicated by black squares on the corresponding topographic maps. Note: Shaded time windows indicate the measurement windows for N170 (150–200 ms), VPP (150–250 ms), and P300/LPP complex (320–600 ms)

**Fig. 4:**
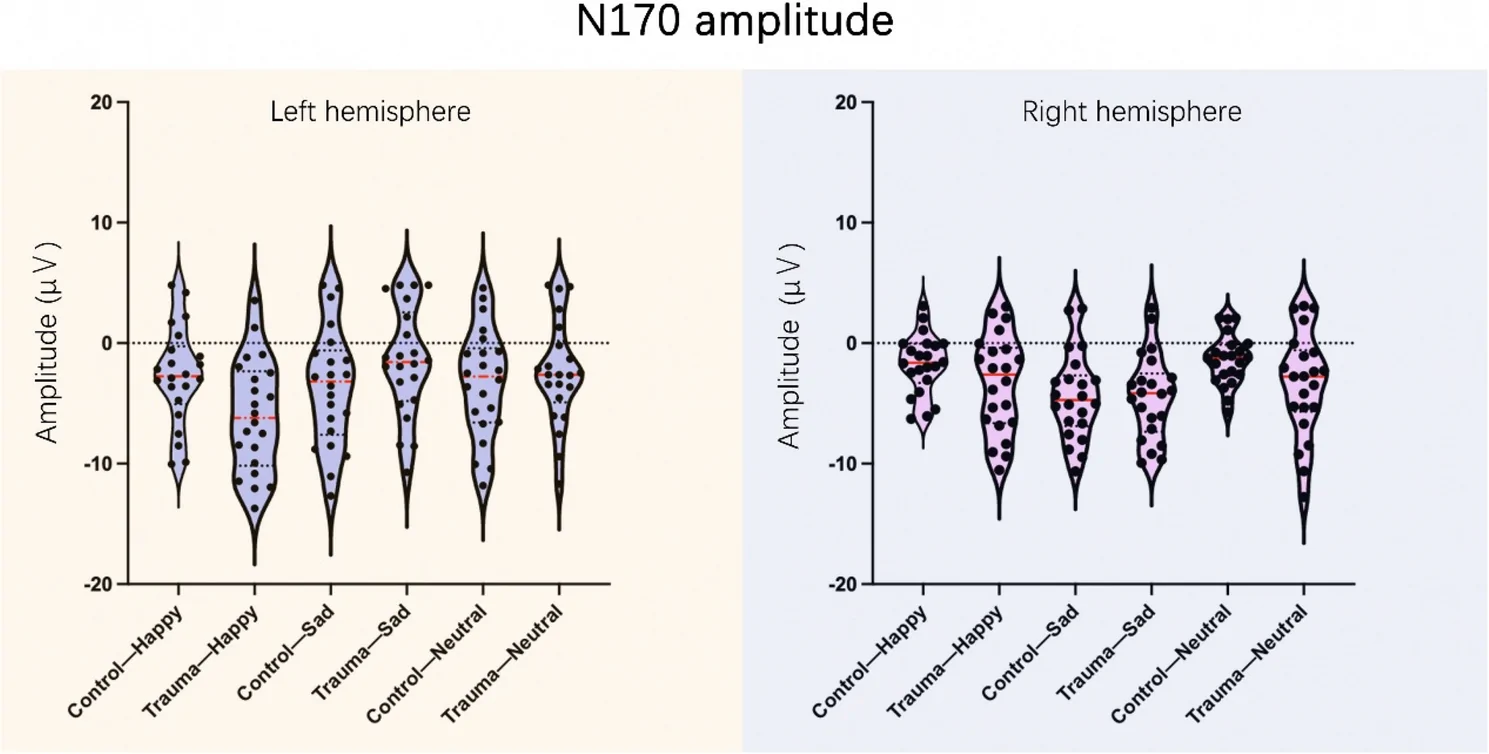
Individual-level distributions of N170 mean amplitudes. Violin plots show N170 mean amplitudes extracted from the 150–200 ms window for the left and right posterior ROIs across group and emotion conditions. Black dots represent individual participants and are jittered horizontally for visibility. Red dashed lines indicate cell means. Violin contours represent smoothed density estimates and may extend slightly beyond the observed data range. Note: Violin contours represent kernel density estimates and may extend slightly beyond the observed data range due to smoothing

Averaged ERP waveforms and scalp topographies of the main ERP components elicited by schematic emotional faces are shown in Fig. 3. Statistical results for ERP amplitudes and latencies are presented in Tables 3 and 4. Individual-level distributions of N170 amplitudes are shown in Fig. 4.

**Table 3:** Summary of statistical analysis of ERP latency

Variables	N170			VPP			P300/LPP complex		
	F	*p*	ηp²	F	*p*	ηp²	F	*p*	ηp²
Group	6.28	.016	.130	9.68	.003	0.187	4.72	.036	.101
Emotion	25.91	< .001	.381	7.22	.001	0.147	12.79	< .001	.233
Hemisphere	15.31	< .001	.267	—	—	—	—	—	—
Group × emotion	0.27	.766	.006	0.04	.966	0.001	13.20	< .001	.239
Group × hemisphere	0.03	.867	.001	—	—	—	—	—	—
Emotion × hemisphere	2.14	.124	.049	—	—	—	—	—	—
Group × hemisphere×emotion	11.49	< .001	.215	—	—	—	—	—	—

Degrees of freedom varied across effects and are reported in the text. Greenhouse–Geisser-corrected results are reported where appropriate. Dashes indicate effects that were not included in the model. Statistical significance was set at *p* < .05

**Table 4:** Summary of statistical analysis of ERP amplitude

Variables	N170			VPP			P300/LPP complex		
	F	*p*	ηp²	F	*p*	ηp²	F	*p*	ηp²
Group	0.29	.593	.007	1.59	0.215	0.036	5.53	.023	.116
Emotion	2.01	.151	.046	7.12	0.004	0.145	0.60	.510	.014
Hemisphere	0.06	.802	.002	—	—	—	—	—	—
Group × emotion	4.03	.031	.088	8.03	0.002	0.160	7.90	.002	.158
Group × hemisphere	1.51	.226	.035	—	—	—	—	—	—
Emotion ×hemisphere	6.22	.008	.129	—	—	—	—	—	—
Group ×hemisphere×emotion	2.06	.148	.047	—	—	—	—	—	—

Degrees of freedom varied across effects and are reported in the text. Greenhouse–Geisser-corrected results are reported where appropriate. Dashes indicate effects that were not included in the model. Statistical significance was set at *p* < .05

### N170

Analysis of N170 amplitudes did not reveal a significant main effect of group, *F(*1, 42) = 0.29, *p* = .593, ηp² = 0.007. The childhood trauma group showed more negative overall N170 amplitudes (*M* = − 3.71 µV, *SD* = 4.24 µV) than the control group (*M = −* 2.77 µV, *SD =* 2.88 µV), but this difference was not statistically significant. Mauchly’s test indicated that the assumption of sphericity was violated for emotion (*W =* 0.728, *p* = .002), and for the emotion-by-hemisphere interaction (*W =* 0.631, *p* < .001). Therefore, Greenhouse–Geisser-corrected results are reported for effects involving emotion. The main effect of emotion was not significant, Greenhouse–Geisser-corrected *F(*1.57, 66.05) = 2.01, *p* = .151, ηp² = 0.046. Bonferroni-adjusted comparisons showed no significant differences among happy faces (*M = −* 3.42 µV, *SD =* 4.24 µV), sad faces (*M = −* 3.79 µV, *SD =* 3.41 µV), and neutral faces (*M = −* 2.51 µV, *SD =* 4.00 µV). Happy and sad faces did not differ significantly (Δ = 0.38 µV, adjusted *p* = 1.000). Happy and neutral faces also did not differ significantly (Δ = −0.91 µV, adjusted *p* = .282). Sad and neutral faces did not differ significantly either (Δ = −1.28 µV, adjusted *p* = .360). The main effect of hemisphere was not significant, *F(*1, 42) = 0.06, *p* = .802, ηp² = 0.002. N170 amplitudes over the left hemisphere (*M = −* 3.32 µV, *SD =* 4.30 µV) did not differ significantly from those over the right hemisphere (*M = −* 3.16 µV, *SD =* 3.53 µV; Δ = −0.16 µV, adjusted *p* = .802). Importantly, the emotion-by-group interaction was significant, Greenhouse–Geisser-corrected *F(*1.57, 66.05) = 4.03, *p* = .031, ηp² = 0.088. Bonferroni-adjusted follow-up comparisons showed that in the childhood trauma group, happy faces (*M = −* 4.81 µV, *SD =* 4.54 µV) did not elicit significantly different N170 amplitudes from sad faces (*M = −* 3.32 µV, *SD =* 4.07 µV; Δ = −1.49 µV, adjusted *p* = .264) or neutral faces (*M = −* 3.01 µV, *SD =* 4.38 µV; Δ = −1.80 µV, adjusted *p* = .062). Sad and neutral faces also did not differ significantly in the childhood trauma group (Δ = −0.31 µV, adjusted *p* = 1.000). In contrast, in the control group, sad faces (*M = −* 4.27 µV, *SD =* 2.60 µV) elicited significantly more negative N170 amplitudes than happy faces (*M = −* 2.03 µV, *SD =* 3.48 µV; Δ = −2.24 µV, adjusted *p* = .036). The difference between sad and neutral faces (*M = −* 2.01 µV, *SD =* 3.63 µV; Δ = −2.26 µV, adjusted *p* = .165) was not significant. Happy and neutral faces did not differ significantly in the control group (Δ = −0.02 µV, adjusted *p* = 1.000). The hemisphere-by-group interaction was not significant, *F(*1, 42) = 1.51, *p* = .226, ηp² = 0.035. Thus, the hemispheric distribution of N170 amplitudes did not differ significantly between the childhood trauma group and the control group. The emotion-by-hemisphere interaction was significant, Greenhouse–Geisser-corrected *F(*1.46, 61.36) = 6.22, *p* = .008, ηp² = 0.129. Bonferroni-adjusted follow-up comparisons showed that happy faces elicited more negative N170 amplitudes over the left hemisphere (*M = −* 4.48 µV, *SD =* 4.85 µV) than over the right hemisphere (*M = −* 2.36 µV, *SD =* 4.01 µV; Δ = −2.11 µV, adjusted *p* = .007). For sad faces, the difference between the left hemisphere (*M = −* 2.62 µV, *SD =* 4.84 µV) and the right hemisphere (*M = −* 4.97 µV, *SD =* 4.46 µV) was not significant after correction, Δ = 2.34 µV, adjusted *p* = .094. For neutral faces, N170 amplitudes did not differ significantly between the left hemisphere (*M = −* 2.86 µV, *SD =* 4.40 µV) and the right hemisphere (*M = −* 2.16 µV, *SD =* 4.10 µV), Δ = −0.71 µV, adjusted *p* = .291. Finally, the emotion-by-hemisphere-by-group interaction was not significant, Greenhouse–Geisser-corrected *F(*1.46, 61.36) = 2.06, *p* = .148, ηp² = 0.047. Therefore, no further interpretation of the three-way interaction was warranted.

Analysis of N170 latency revealed a significant main effect of group, *F*(1, 42) = 6.28, *p* = .016, ηp² = 0.130, with the control group showing longer N170 latencies (*M* = 177.06ms, *SD* = 9.53ms) than the childhood trauma group (*M* = 170.27ms, *SD* = 8.40ms). The main effect of emotion was significant, *F*(2, 84) = 25.91, *p* < .001, ηp² = 0.381. Bonferroni-adjusted comparisons showed that sad faces (*M* = 169.51ms, *SD* = 9.27ms) elicited shorter N170 latencies than happy faces (*M* = 176.47 ms, *SD* = 10.21 ms; Δ = −6.96 ms, *p* < .001) and neutral faces (*M* = 175.01 ms, *SD* = 11.26 ms; Δ = −5.50 ms, *p* < .001), whereas happy and neutral faces did not differ significantly (Δ = 1.46ms, *p* = .680). A significant main effect of hemisphere was also observed, *F*(1, 42) = 15.31, *p* < .001, ηp² = 0.267, indicating longer latencies over the left hemisphere (*M* = 175.56 ms, *SD* = 9.61 ms) than over the right hemisphere (*M* = 171.77 ms, *SD* = 10.45 ms). The emotion-by-group interaction was not significant, *F*(2, 84) = 0.27, *p* = .766, ηp² = 0.006, nor was the hemisphere-by-group interaction, *F*(1, 42) = 0.03, *p* = .867, ηp² = 0.001. However, the emotion-by-hemisphere-by-group interaction was significant, *F*(2, 84) = 11.49, *p* < .001, ηp² = 0.215, indicating that emotional modulation of N170 latency varied as a function of both hemisphere and group. Full statistics are reported in Tables 3 and 4.

### VPP

Analysis of VPP amplitudes revealed no significant main effect of group, *F*(1, 42) = 1.59, *p* = .215, ηp² = 0.036, with no reliable overall amplitude difference between the control group (*M* = 3.32µV, *SD* = 1.25µV) and the childhood trauma group (*M* = 3.75µV, *SD* = 0.99µV). Mauchly’s test indicated that the assumption of sphericity was violated for emotion. Therefore, Greenhouse–Geisser-corrected results are reported for effects involving emotion. The main effect of emotion was significant, Greenhouse–Geisser-corrected *F*(1.54, 64.85) = 7.12, *p* = .004, ηp² = 0.145. Bonferroni-adjusted comparisons showed that happy faces (*M* = 4.55 µV, *SD* = 2.51 µV) elicited larger VPP amplitudes than sad faces (*M* = 3.16 µV, *SD* = 1.99 µV; Δ = 1.39 µV, *p* = .004) and neutral faces (*M* = 2.90 µV, *SD* = 2.22 µV; Δ = 1.65 µV, *p* = .022), whereas sad and neutral faces did not differ significantly (Δ = 0.26 µV, *p* = 1.000). The emotion-by-group interaction was significant, Greenhouse–Geisser-corrected *F*(1.54, 64.85) = 8.03, *p* = .002, ηp² = 0.160. Bonferroni-adjusted p values are reported for all follow-up comparisons. Follow-up Bonferroni-adjusted simple comparisons were conducted to further examine the group × emotion interaction for VPP mean amplitudes. Descriptive cell means showed that, in the control group, VPP amplitudes were 3.41 µV for happy faces (SD = 2.50 µV), 2.91 µV for sad faces (SD = 2.05 µV), and 3.65 µV for neutral faces (SD = 2.21 µV). In the childhood trauma group, VPP amplitudes were 5.69 µV for happy faces (SD = 1.98 µV), 3.41 µV for sad faces (SD = 1.95 µV), and 2.15 µV for neutral faces (SD = 2.00 µV). Group comparisons within each emotion showed that the childhood trauma group had larger VPP amplitudes than the control group for happy faces, Δ = 2.28 µV, adjusted *p* = .002. No significant group difference was observed for sad faces, Δ = 0.50 µV, adjusted *p* = .413. For neutral faces, the control group showed larger VPP amplitudes than the childhood trauma group, Δ = 1.49 µV, adjusted *p* = .024. Within-group emotion comparisons showed that no pairwise comparison reached significance in the control group after Bonferroni correction: happy versus sad, Δ = 0.50 µV, adjusted *p* = 1.000; happy versus neutral, Δ = −0.23 µV, adjusted *p* = 1.000; and sad versus neutral, Δ = −0.74 µV, adjusted *p* = .599. In the childhood trauma group, happy faces elicited larger VPP amplitudes than sad faces, Δ = 2.28 µV, adjusted *p* = .001, and neutral faces, Δ = 3.53 µV, adjusted *p* < .001. The sad–neutral comparison in the childhood trauma group was not significant after correction, Δ = 1.26 µV, adjusted *p* = .094.

Analysis of VPP latency revealed a significant main effect of group, *F*(1, 42) = 9.68, *p* = .003, ηp² = 0.187, with the childhood trauma group showing longer VPP latencies (*M* = 188.14 ms, *SD* = 12.57 ms) than the control group (*M* = 176.17 ms, *SD* = 12.94 ms). The main effect of emotion was also significant, *F*(2, 84) = 7.22, *p* = .001, ηp² = 0.147. Bonferroni-adjusted comparisons showed that neutral faces (*M* = 187.95 ms, *SD* = 18.96 ms) elicited longer VPP latencies than happy faces (*M* = 176.25 ms, *SD* = 17.84 ms; Δ = 11.71 ms, *p* = .003). Happy faces did not differ significantly from sad faces (*M* = 182.25 ms, *SD* = 17.27 ms; Δ = 6.00 ms, *p* = .157), and sad and neutral faces did not differ significantly (Δ = 5.71 ms, *p* = .160). The emotion-by-group interaction was not significant, *F*(2, 84) = 0.04, *p* = .966, ηp² = 0.001. Descriptively, the childhood trauma group showed longer VPP latencies than the control group across happy faces (childhood trauma group: *M* = 182.00 ms, *SD* = 19.83 ms; controls: *M* = 170.50 ms, *SD* = 12.36 ms), sad faces (trauma: *M* = 188.00 ms, *SD* = 14.24 ms; controls: *M* = 176.50 ms, *SD* = 19.04 ms), and neutral faces (childhood trauma group: *M* = 194.41 ms, *SD* = 19.62 ms; controls: *M* = 181.50 ms, *SD* = 17.77 ms). Full statistics are reported in Tables 3 and 4.

### P300/LPP complex

Analysis of P300/LPP complex amplitudes revealed a significant main effect of group, *F(*1, 42) = 5.53, *p* = .023, ηp² = 0.116, with larger overall P300/LPP complex amplitudes in the childhood trauma group (*M =* 5.37 µV, *SD =* 3.26 µV) than in the control group (*M =* 2.96 µV, *SD =* 1.93 µV). Mauchly’s test indicated that the assumption of sphericity was violated for emotion, *W =* 0.686, χ²(2) = 15.45, *p* < .001, εGG = 0.761. Therefore, Greenhouse–Geisser-corrected results are reported for effects involving emotion. Bonferroni adjustment was used for follow-up pairwise comparisons. The main effect of emotion was not significant, Greenhouse–Geisser-corrected *F(*1.52, 63.93) = 0.60, *p* = .510, ηp² = 0.014. Bonferroni-adjusted comparisons showed no significant differences among happy faces (*M =* 4.02 µV, *SD =* 3.74 µV), sad faces (*M =* 4.67 µV, *SD =* 3.26 µV), and neutral faces (*M =* 3.82 µV, *SD =* 3.25 µV). Happy and sad faces did not differ significantly (Δ = −0.65 µV, adjusted *p* = .929). Happy and neutral faces also did not differ significantly (Δ = 0.20 µV, adjusted *p* = 1.000). Sad and neutral faces did not differ significantly either (Δ = 0.85 µV, adjusted *p* = .796). The emotion-by-group interaction was significant, Greenhouse–Geisser-corrected *F(*1.52, 63.93) = 7.90, *p* = .002, ηp² = 0.158. Bonferroni-adjusted simple-effects analyses showed that in the childhood trauma group, sad faces elicited the largest P300/LPP complex amplitudes (*M =* 7.00 µV, *SD =* 2.62 µV), followed by neutral faces (*M =* 5.73 µV, *SD =* 2.89 µV) and happy faces (*M =* 3.38 µV, *SD =* 4.27 µV). Sad faces elicited significantly larger amplitudes than happy faces in the childhood trauma group (Δ = 3.62 µV, adjusted *p* = .001). However, the differences between sad and neutral faces (Δ = 1.27 µV, adjusted *p* = .716) and between happy and neutral faces (Δ = −2.35 µV, adjusted *p* = .316) were not significant. In contrast, in the control group, happy faces elicited the largest P300/LPP complex amplitudes (*M =* 4.66 µV, *SD =* 3.10 µV), compared with sad faces (*M =* 2.33 µV, *SD =* 1.85 µV) and neutral faces (*M =* 1.90 µV, *SD =* 2.36 µV). Happy faces elicited significantly larger amplitudes than sad faces in the control group (Δ = 2.33 µV, adjusted *p* = .037). The difference between happy and neutral faces was not significant (Δ = 2.75 µV, adjusted *p* = .179), and sad and neutral faces also did not differ significantly (Δ = 0.43 µV, adjusted *p* = 1.000). Group comparisons within each emotion showed that the childhood trauma group had significantly larger P300/LPP complex amplitudes than the control group for sad faces (childhood trauma: *M =* 7.00 µV, *SD =* 2.62 µV; control: *M =* 2.33 µV, *SD =* 1.85 µV; Δ = 4.67 µV, adjusted *p* = .001) and neutral faces (childhood trauma: *M =* 5.73 µV, *SD =* 2.89 µV; control: *M =* 1.90 µV, *SD =* 2.36 µV; Δ = 3.83 µV, adjusted *p* < .001). The group difference for happy faces was not significant (childhood trauma: *M =* 3.38 µV, *SD =* 4.27 µV; control: *M =* 4.66 µV, *SD =* 3.10 µV; Δ = −1.28 µV, adjusted *p* = .502).

Analysis of P300/LPP complex latency revealed a significant main effect of group, *F*(1, 42) = 4.72, *p* = .036, ηp² = 0.101, with the childhood trauma group (*M* = 433.70 ms, *SD* = 25.83 ms) showing longer overall P300/LPP complex latencies than the control group (*M* = 414.47 ms, *SD* = 32.50 ms). Mauchly’s test indicated that the assumption of sphericity was violated for emotion. Therefore, Greenhouse–Geisser-corrected results are reported. The main effect of emotion was significant, Greenhouse–Geisser-corrected *F*(1.71, 71.81) = 12.79, *p* < .001, ηp² = 0.233. Bonferroni-adjusted comparisons showed that happy faces (*M* = 427.00 ms, *SD* = 26.73 ms) elicited longer P300/LPP complex latencies than sad faces (*M* = 405.37 ms, *SD* = 29.16 ms; Δ = 21.64 ms, *p* = .007), and neutral faces (*M* = 439.89 ms, *SD* = 27.77 ms) also elicited longer latencies than sad faces (Δ = 34.52 ms, *p* < .001). Happy and neutral faces did not differ significantly (Δ = −12.89 ms, *p* = .360). The emotion-by-group interaction was significant, Greenhouse–Geisser-corrected *F*(1.71, 71.81) = 13.20, *p* < .001, ηp² = 0.239. Group comparisons within each emotion showed that the childhood trauma group had longer P300/LPP complex latencies than the control group for happy faces (childhood trauma: *M* = 453.09 ms, *SD* = 20.39 ms; controls: M = 400.91 ms, *SD* = 29.44 ms; Δ = 52.18 ms, *p* = .001) and neutral faces (childhood trauma group: *M* = 451.77 ms, *SD* = 21.85 ms; controls: *M* = 428.00 ms, *SD* = 29.57 ms; Δ = 23.77 ms, *p* = .035). In contrast, the control group showed longer latencies than the childhood trauma group for sad faces (controls: *M* = 414.50 ms, *SD* = 23.62 ms; childhood trauma: *M* = 396.23 ms, *SD* = 20.89 ms; Δ = 18.27 ms, *p* = .036). Full statistics are reported in Tables 3 and 4.

## Discussion

Previous research has suggested that childhood trauma may be associated with alterations in the processing of emotional facial expressions, which could contribute to the onset and maintenance of later psychopathology. Research findings in this area remain inconsistent. Some studies reported faster responses to fearful expressions in maltreated children [6], whereas others found slower behavioral responses in individuals with childhood adversity [24]. These differences likely reflect differences in methods across studies. Studies often varied in sample characteristics and task demands, and particularly the type and standardization of facial stimuli. Given these inconsistencies, this study used an emotion classification task with schematic facial stimuli. We examined how childhood trauma affects both behavioral and neural processes involved in facial emotion recognition.

Behaviorally, individuals with childhood trauma showed significantly longer overall reaction times than controls. This pattern should not be interpreted as a speed–accuracy trade-off, because the slower responses were not accompanied by higher accuracy. Instead, the trauma group may have required more time to evaluate and classify schematic emotional facial configurations while maintaining comparable overall accuracy. This pattern can be understood within the framework of latent vulnerability [13], which proposes that childhood maltreatment recalibrates neurocognitive systems involved in threat detection and social-information processing. Such recalibration may increase evaluative demands during the processing of emotional facial configurations, thereby reducing classification efficiency without impairing accuracy. This interpretation is also broadly consistent with the distinction between processing efficiency and performance effectiveness proposed in Attentional Control Theory [30], although that framework was originally developed in the context of anxiety. Most prior research on childhood adversity and facial emotion recognition has focused primarily on recognition accuracy across selected emotional categories, whereas reaction time differences have received less systematic attention [2, 24]. As a result, direct comparisons with the present reaction time findings are limited. At the emotion-specific level, the trauma group responded more slowly than controls to happy and sad faces, but not to neutral faces. This pattern suggests that their processing is emotion-dependent: when stimuli carry a clear positive or negative valence, classification becomes more time-consuming, whereas processing of relatively affectively “minimal” neutral faces is spared. Within-group analyses further showed that, for the trauma group, reaction times were longest for happy faces, followed by sad faces, and shortest for neutral faces. This hierarchy partly aligns with the traditional threat vigilance model, which posits that trauma heightens automatic attention to negative or threat-related cues such as sad or angry expressions [31]. In particular, the finding that sad faces elicited slower responses than neutral faces in the trauma group is consistent with this account and with previous studies documenting longer reaction times to negative expressions among maltreated children [24]. At the same time, our observation that responses to happy faces were even more delayed than responses to sad faces goes beyond what the traditional threat vigilance model would predict, suggesting that additional mechanisms are likely to be involved. One plausible candidate is that childhood trauma has been robustly linked to anhedonia and broader difficulties in the processing of positive affect [32]. These alterations may extend from subjective experience to cognitive operations on external positive signals, such that when individuals with trauma are confronted with a positive facial expression that does not match their internal affective state, they require more time to categorize and interpret the stimulus. The present within-group analyses suggest that reaction time differences in childhood trauma samples may vary systematically across emotion categories, with effects on positive expressions potentially as pronounced as or exceeding those on negative ones. This pattern has received limited attention in prior work, which has seldom directly compared responses to happy and sad faces within the same trauma sample, and it points to the value of examining the full valence spectrum rather than focusing exclusively on negative or threat-relevant stimuli.

The ERP results provide temporal evidence that childhood trauma was associated with differences in neural responses during schematic emotional face processing. The N170 was examined as a posterior ERP component commonly used to characterize early face-sensitive processing [9, 20]. In the present study, the N170 findings concerned the timing of this posterior response and its emotion-specific amplitude modulation. For N170 latency, the latency results showed earlier N170 peak timing in the childhood trauma group than in the control group. The latency results showed earlier N170 peak timing in the childhood trauma group than in the control group. This study found that childhood trauma was associated with an earlier posterior N170 peak across schematic facial configurations, suggesting altered temporal readiness for registering simplified facial cues at an early face-sensitive processing stage. Direct evidence on N170 latency in childhood trauma or maltreatment samples remains limited, so the closest comparisons come from deprivation-related studies of face processing. In these studies, severe institutional rearing or psychosocial deprivation has been associated with delayed or atypical N170 latencies; some studies found no clear N170 latency difference between groups [33, 34]. The present study showed a different latency pattern, with shorter overall N170 latency in adults with retrospective childhood trauma. One possible source of this difference is that deprivation-based studies and the present study capture different forms of early adverse experience. Institutional deprivation involves marked reductions in expected social and sensory input during development, whereas the present study assessed childhood trauma histories. Dimensional models distinguishing threat from deprivation suggest that different forms of adversity may shape social-information processing through partly distinct pathways [35]. Consistent with this view, maltreatment-related evidence indicates that adverse interpersonal experience can alter attention and responsiveness to emotionally salient facial cue [31, 36]. In the present task, shorter N170 latency may reflect heightened temporal readiness to register schematic socially relevant cues at the posterior face-sensitive stage. For N170 amplitude, the results did not follow the hypothesized pattern of stronger negative-emotion modulation in the childhood trauma group. The significant Group × Emotion interaction reflected sad–happy differentiation in the control group, with no comparable emotion-specific differentiation in the childhood trauma group. The absence of significant group and emotion main effects further indicates that this pattern was not driven by a general enhancement or attenuation of N170 amplitude in the childhood trauma group. Prior ERP studies show that facial expressions can influence N170 amplitude within the early face-processing window [37, 38]. Schematic-face evidence further indicates that simplified emotional facial configurations can modulate N170 responses when emotional meaning is explicitly processed [39]. This evidence links N170 amplitude to the question of whether childhood trauma is associated with altered early emotion differentiation during facial emotion processing. The original prediction was that childhood trauma would be associated with stronger N170 modulation for negative schematic facial configurations than for happy or neutral configurations. The observed Group × Emotion pattern points to a different form of trauma-related early emotion modulation. Rather than amplified responses to sad schematic faces, the childhood trauma group showed reduced or absent sad–happy differentiation at the early posterior N170 amplitude level. A more plausible interpretation is that childhood trauma may alter the specificity of early emotion differentiation. In the control group, the sad–happy difference in N170 amplitude suggests that negative and positive schematic facial cues were distinguished at the early posterior N170 stage. In the childhood trauma group, the lack of evidence is consistent with latent vulnerability models proposing that adverse caregiving experience can recalibrate neurocognitive systems involved in processing socially salient information, with heightened responsiveness in some contexts and reduced or avoidant responsiveness in others [13, 14]. The absence of predicted sad-related N170 amplification in the childhood trauma group may reflect the emotion-specific nature of maltreatment-related face processing. Behavioral and attentional work on maltreatment more often reports pronounced effects for direct threat cues such as anger or fear than for negative expressions as a general category [40, 41]. Sad schematic faces convey negative interpersonal information, but they do not provide the same direct threat signal as anger or fear. In the present task, this may explain why childhood trauma was not associated with amplified early posterior responses to sad schematic faces. The Group × Emotion interaction instead reflected sad–happy N170 differentiation in the control group and reduced or absent differentiation in the childhood trauma group, suggesting that childhood trauma was associated with altered early emotion differentiation rather than simple negative-emotion amplification. The N170 amplitude findings suggest that childhood trauma was associated with a different specificity of early emotion differentiation, with sad-related processing not emerging as a simple early posterior enhancement in this task context. The later P300/LPP findings showed a different descriptive pattern, with sad-related weighting more evident in the childhood trauma group during later evaluative processing.

The VPP was examined as a fronto-central index of early face-related neural activity within the same early time window as the posterior N170 [17]. Direct evidence linking childhood trauma to VPP responses during facial emotion recognition remains limited, so the VPP findings were interpreted as evidence of group-dependent emotional modulation rather than within a strong directional prediction. For VPP amplitude, the Group × Emotion interaction indicated a condition-specific modulation of VPP amplitude. At the VPP stage, emotional facial information can modulate the response, although specific expressions are not consistently differentiated within this early time window [42]. This background helps locate the present Group × Emotion interaction as happy-centered VPP modulation in the childhood trauma group, rather than as general VPP sensitivity to emotional faces. This happy-centered pattern may be understood in light of evidence that maltreatment-related early face processing is not restricted to threat-related expressions. Across attentional and ERP studies, maltreatment-related differences in early face processing have involved emotional faces relative to neutral faces as well as positive facial affect, placing the present happy-centered VPP modulation within evidence that these effects are not restricted to threat-related cues [36, 43]. At the level of peak timing, the childhood trauma group showed longer overall VPP latencies than the control group. Direct evidence on VPP latency in individuals with childhood trauma histories remains limited, but this group-level delay suggests that early face-related activity in the VPP window followed a later time course in the childhood trauma group. Because the Group × Emotion interaction was not significant, this delay was not tied to a specific schematic emotional configuration. Across both groups, neutral schematic faces showed longer VPP latencies than happy faces, indicating a group-general timing difference rather than a trauma-specific latency effect. For VPP latency, the childhood trauma group showed longer overall latencies than the control group. This longer latency reflects a later temporal peak of early face-related VPP activity in the childhood trauma group during schematic emotional face processing. Empirical evidence from adults with childhood maltreatment histories has linked maltreatment to altered facial emotion recognition, including timing-related differences in responses to emotional expressions [2]. The present latency finding extends this evidence to the early VPP window by showing a later-developing face-related response in the childhood trauma group. Neutral schematic faces also elicited longer VPP latencies than happy faces across participants, indicating a group-general category effect on VPP peak timing.

Childhood trauma was associated with a control-referenced reconfiguration of early face-sensitive activity within the N170, VPP window. This interpretation rests on the temporal overlap and close relation between the N170 and VPP as scalp-recorded indices of early face processing [17]. In the control group, emotional differentiation in this window was expressed as posterior N170 sad–happy differentiation. In the childhood trauma group, this posterior profile was not preserved; emotion weighting appeared instead as happy-dominant fronto-central VPP modulation. The latency results showed a similar departure from a single early-window direction, with earlier N170 but later VPP peak timing in the childhood trauma group. Early ERP studies of emotional face processing distinguish structural encoding of faces from expression analysis, with emotional-expression effects sometimes emerging early even when the face-sensitive N170 is not modulated by expression [44]. This distinction helps frame the present profile as a shift in the relative expression of early structural and emotional-cue processing within the N170, VPP window. Maltreatment research provides a more specific basis for the present group pattern. Early adverse experience can alter the perceptual organization and neural prioritization of emotional facial cues, with ERP studies showing selective and shifted affective-face responses relative to nonmaltreated controls [1, 5, 41]. From this perspective, the present N170, VPP profile is best characterized as experience-dependent reweighting of early emotional-face processing, with reduced or absent posterior sad–happy differentiation and happy-weighted VPP activity in the childhood trauma group. Maltreatment-related changes in face processing take multiple cue-specific forms rather than a single emotional-bias pattern. Childhood maltreatment has been associated with altered attention to angry faces, faster recognition of fearful faces, threat avoidance under abuse- or PTSD-related conditions, enhanced responses to ambiguous threat, negativity bias for neutral expressions, and heightened perceived intensity of happy faces [6, 40, 45]. This evidence supports a cue-specific account of the present N170,VPP profile, in which happy-weighted VPP activity and reduced or absent posterior sad–happy differentiation reflect altered early social salience rather than a simple preference for happy faces or a uniform threat-vigilance pattern.

The late positive response in the 320–600 ms window was treated as a P300/LPP complex because this interval overlaps with the classical P300 window and the early portion of the late positive potential, two late positive responses with closely related empirical and theoretical profiles [46]. Within this broader P300/LPP framework, late positive activity is linked to stimulus evaluation, attentional engagement, and sustained processing of emotionally and motivationally significant information, making it relevant to later evaluative processing of schematic emotional facial configurations [47, 48]. The observed amplitude pattern differed from the predicted reduction in late positive responses to negative emotional faces in the childhood trauma group. Instead, the childhood trauma group showed larger P300/LPP amplitudes for sad and neutral schematic faces, with no corresponding group difference for happy faces. Thus, the present P300/LPP pattern aligns more closely with a competing account in which childhood trauma is associated with enhanced late evaluative weighting of negative or interpersonally uncertain cues, rather than with attenuated later positive activity. This account is consistent with maltreatment-related evidence showing heightened late positive responses to salient negative or ambiguous threat-related facial information, while also recognizing that reduced late positive responses have been reported in other adverse caregiving or trauma-related contexts. The childhood trauma group showed longer overall P300/LPP latencies than the control group, consistent with a later temporal peak of the late positive response during schematic emotional face processing. Happy and neutral schematic faces showed later P300/LPP peaks in the childhood trauma group, whereas sad faces showed the opposite group difference. The combined amplitude and latency pattern suggests that sad schematic facial configurations may have carried greater late-evaluative significance for the childhood trauma group than for controls. This interpretation fits with maltreatment-related evidence that adverse caregiving experiences can tune attention and evaluation toward salient negative interpersonal cues. Maltreatment studies link late positive activity to selective allocation of attention toward salient negative or threat-related facial information. Enhanced late positive activity to angry target faces in maltreated children has been interpreted as altered allocation of attention to negative facial cues [18], and stronger LPP responses to increasingly fearful facial expressions in adults with childhood maltreatment histories have been interpreted as heightened sensitivity to subtle threat-related content [49]. Sad schematic faces were not direct threat cues in the same way as anger or fear, but they may have carried interpersonal meanings related to rejection, distress, or social disengagement. The larger and earlier-peaking P300/LPP response to sad faces may therefore reflect enhanced late-evaluative prioritization of negative interpersonal cues in the childhood trauma group.

The present study adds to existing research by examining childhood trauma-related differences in the processing of schematic emotional facial configurations with both behavioral and time-resolved ERP measures. The ERP findings revealed trauma-related differences in the timing and emotional weighting of schematic face processing. Within the early face-sensitive window, the N170 and VPP results did not show a parallel timing pattern, with earlier N170 peak timing but later VPP peak timing in the childhood trauma group. The later P300/LPP results further showed a larger overall and temporally altered late positive response in the childhood trauma group, with group-dependent emotional weighting. These findings indicate that childhood trauma was associated with altered timing and cue-specific prioritization during schematic emotional face processing, rather than a uniform change in face-related responsiveness. This pattern was evident both within the early face-sensitive window, where N170 and VPP timing did not move in parallel, and across processing windows, where later P300/LPP activity showed larger and temporally altered responses in the childhood trauma group. Future work can build on this approach by examining how these ERP profiles relate to symptom dimensions, interpersonal functioning, and behavioral indices of emotion evaluation.

Several limitations of this study should be acknowledged. The use of schematic facial configurations, while effective for isolating the contribution of configural cues and eliminating confounds associated with identity, gender, and ethnicity, limits the ecological validity of the findings. Schematic faces convey emotion primarily through mouth curvature, whereas naturalistic expressions involve coordinated changes across multiple facial features. With only three stimulus exemplars, participants may have partially relied on simple curvature detection rather than holistic emotion recognition. However, if the task had been reduced entirely to curvature detection, the stage-dependent and emotion-specific ERP patterns observed, including opposite emotion effects between groups at the N170 and VPP stages, would be difficult to account for solely on the basis of low-level visual discrimination. Nevertheless, the possibility that configural simplicity partially shifted processing toward feature-level detection cannot be excluded, and future studies should consider including a wider range of schematic configurations to reduce stimulus predictability. Second, The use of three facial expression categories is not unusual in childhood maltreatment research. Prior ERP studies in maltreated children have used three-category stimulus sets, typically angry, happy, and neutral faces [1, 43], and related neuroimaging work in early life maltreatment has used happy, sad, and neutral facial expressions [50]. Although this selection provided a focused contrast among positive, negative, and neutral facial expressions, it limits the generalizability of the observed effects and prevents examination of whether the trauma-related processing alterations extend to emotions frequently implicated in trauma research, such as fear or anger. Future work incorporating a broader set of emotional expressions would help clarify the specificity and boundaries of the present findings. Third, no inverted or non-face control stimuli were included. Without such conditions, the degree to which the observed ERP effects reflect face-specific processing rather than responses to basic visual configurations cannot be fully established. The scalp distributions and temporal characteristics of the components examined were consistent with those reported in the face-processing literature, but including inverted or scrambled control stimuli in future designs would provide more direct evidence for face-sensitivity. Finally, the study employed a cross-sectional design, which precludes causal inference about the relationship between childhood trauma and the observed neural processing patterns. Longitudinal designs would help clarify how early adversity influences the development of these processing characteristics over time.

## Conclusion

This study examined childhood trauma related differences in the processing of schematic emotional facial configurations using behavioral and time resolved ERP measures. Childhood trauma history was associated with slower behavioral responses and with ERP differences across the early N170, VPP and later P300/LPP windows. In the early window, the childhood trauma group showed earlier N170 peak timing but later VPP peak timing. In the later P300/LPP window, the childhood trauma group showed larger overall late positive responses, with sad faces eliciting larger amplitudes than happy faces, whereas the control group showed the opposite sad–happy pattern. These findings indicate that childhood trauma was associated with differentiated timing and evaluative responses to schematic emotional facial configurations, rather than a uniform enhancement or attenuation of face-related responsiveness.

## Data Availability

The datasets used and/or analyzed during the current study are available from the corresponding author on reasonable request.
